# On the Production of Hæmorrhage, Anæmia, Œdema, and Emphysema in the Lungs by Injuries to the Base of the Brain

**Published:** 1871-05

**Authors:** C. E. Brown-Sequard


*On the Production of Hæmorrhage, Anæmia, Œdema, and Emphysema in the Lungs by Injuries to the Base of the Brain.* By C. E. Brown-Sequard, M. D., &c.

[ From the London Lancet, Jan. 7.]

---

My object in this short paper is to call the attention of practitioners to some experimental facts which, in connexion with a great many clinical facts, show how frequently the lungs are altered consecutively to a lesion of the brain. In making experiments on the comparative lethality of injuries to the left and the right sides of the brain, I found, a year ago, that one of the most frequent causes of death, when it does not occur immediately or very soon after wounds of the brain, in guinea-pigs especially, was pneumonia. I was led by this fact to perform a large number of experiments to ascertain the immediate effects of an injury to the brain on the lungs. The results obtained were startling; in almost all cases of injuries by crushing or section of the pons Varolii, ecchymoses were found in the lungs. Sometimes the whole lung was crowded with effused blood, and real pulmonary apoplexy existed. In some instances the effusion took place in the bronchial tubes. Injuries to other parts of the base of the brain, especially the crura cerebri and the crura cerebelli, sometimes are followed by the same effects on the lung, and it is extremely probable that a slight pressure upon the pons Varolii by effused blood is sufficient to produce it. Injuries to the medulla oblongata and to the spinal cord have but very rarely (only in three or four experiments out of a great many) caused an effusion of blood in the lungs. This is the more remarkable that, without any doubt, the nerve-fibres going from the pons Varolii to the lung, which cause the rupture of small blood-vessels in this viscus, pass through the medulla oblongata and the cervical part of the spinal cord. Many experiments have shown me that it is not through the par vagum, but through the sympathetic nerve, especially by its spinal roots, which throw themselves in the first thoracic ganglion, that the peculiar influence of the irritated pons Varolii exerts itself in producing a pulmonary hæmorrhage.

Many experiments, some of them made with the help of an ingenious physiologist, Dr. J. S. Lombard, of Boston, U. S., have shown me that the condition of the lung, as regards distension or collapse of the air-cells, does not materially change the effect produced on the lungs by the crushing or a wound of the pons Varolii. I have filled the lungs (the thorax opened sometimes) with as much air as insufflation could push in, and seen ecchymoses, small or large, appear at once, or almost at once, after the irritation of the pons. On the other hand, I have withdrawn as much as I could the air contained in the lungs, and ascertained that ecchymoses appeared then, as in the preceding experiments, from the same cause. It is not essential at all that there be a continuation of breathing after an injury to the pons for the production of hæmorrhage in the lungs. Indeed, in all the cases of crushing, and in a great many of the cases of section, of the pons Varolii which I have performed, the kind of syncope which I have described as the respiratory syncope (inhibition of respiration) exists at once after the lesion; and notwithstanding this complete cessation of respiratory movements, the breaking of small blood-vessels takes place in the lungs.

A lesion in one of the lateral halves of the pons produces generally a much greater effect in the lung on the opposite side than in the one on the same side.

The above experiments have been made on guinea-pigs; but in two rabbits and three cats I have found that the section or the crushing of the pons Varolii produced also a hæmorrhage in the lungs.

A hæmorrhage is not the only immediate effect that can be observed after an irritation of the base of the brain by crushing or cutting: an anæmic condition, œdema, and emphysema can also be produced. Some small parts of the lungs are found perfectly white, and, according to the examination of a distinguished micrographer, M. Rauvier, who has kindly helped me in some of these researches, absolutely deprived of blood, no doubt through a spasm of the blood-vessels, having emptied them of their contents. This may occur after injuries of almost all the parts of the base of the brain, but especially the pons Varolii. Not so as regards œdema, which principally appears after an injury to the medulla oblongata. Looking at a lung in which such an alteration exists, one observes one or several greyish spots, generally circular, and of the size of the head of a pin, protruding as a part of a sphere from the surface of the lung. This pearl-like part of the lung, according to my able friend, M. Rauvier, contains a good deal of serum, and its minute blood-vessels are filled with the white corpuscles of blood, and free from red corpuscles. This is, indeed, a most wonderful fact, and the more so that this change in the contents of the pulmonary capillaries is immediate.

The last effect I intend to mention of an injury to the base of the brain on the lungs is already known to experimenters; I mean emphysema. But what I will state about it is a new and very remarkable fact, which does not agree with the reigning theories of the mode of production of emphysema. It is that this morbid condition can appear when not a single respiratory movement takes place, after an irritation of the base of the brain either by crushing or cutting.

When I publish the details of my experiments on the influence of injuries to the brain on the lungs, I will show that, in man, diseases of or injuries to the brain very frequently produce organic alterations in the lungs. I will content myself here, to prove the frequency of that morbid influence of the brain on the pulmonary organs, to state that, out of 188 cases of organic disease of the brain recorded by Calmeil,* there was a morbid state of the lungs, especially inflammation, in more than sixty cases—i. e., in one case out of three. I have no doubt that many patients attacked with brain diseases die from a disease of the lungs caused by that of the central organ of the nervous system.

*January*, 1871.

